# Effects of Ncl. Basalis Meynert volume on the Trail‐Making‐Test are restricted to the left hemisphere

**DOI:** 10.1002/brb3.421

**Published:** 2015-12-29

**Authors:** Florian Lammers, Arian Mobascher, Francesco Musso, Nadim Jon Shah, Tracy Warbrick, Laszlo Zaborszky, Georg Winterer

**Affiliations:** ^1^ Department of Anaesthesiology and Surgical Intensive Care MedicineCharité – University Medicine BerlinBerlinGermany; ^2^ Department of PsychiatryUniversity Hospital MainzMainzGermany; ^3^ Department of PsychiatryHeinrich‐Heine UniversityDüsseldorfGermany; ^4^ Institute of Neuroscience & MedicineResearch Centre JülichJülichGermany; ^5^ Department of NeurologyFaculty of MedicineRWTH Aachen UniversityAachenGermany; ^6^ Centre for Molecular and Behavioral NeuroscienceRutgers The State University of New JerseyNewarkNew Jersey07102; ^7^ Experimental and Clinical Research Centre (ECRC)Charité – University Medicine BerlinBerlinGermany; ^8^ Pharmaimage Biomarker Solutions GmbHBiotech Park Berlin‐BuchRobert‐Rössle‐Str. 1013125BerlinGermany

**Keywords:** Acetylcholine, attention, magnetic resonance imaging (MRI), neuroimaging

## Abstract

**Background:**

Cortical acetylcholine released from cells in the basal forebrain facilitates cue detection and improves attentional performance. Cholinergic fibres to the cortex originate from the CH4 cell group, sometimes referred to as the Nucleus basalis of Meynert and the Nucleus subputaminalis of Ayala. The aim of this work was to investigate the effects of volumes of cholinergic nuclei on attention and executive function.

**Methods:**

The volumes of CH4 and CH4p subregions were measured in a subgroup of 38 subjects (33.5 ± 11 years, 20 females) from a population‐based cohort study of smokers and never‐smokers who have undergone additional MR imaging. To define regions of interest, we applied a DARTEL‐based procedure implemented in SPM8 and a validated probabilistic map of the basal forebrain. Attention and executive function were measured with Trail‐Making Test (TMT A+B) and Stroop‐Task.

**Results:**

We found a quadratic effect of the left CH4 subregion on performance of the TMT. Extremely small as well as extremely large volumes are associated with poor test performance.

**Conclusions:**

Our results indicate that a small CH4 volume predisposes for a hypocholinergic state, whereas an extremely large volume predisposes for a hypercholinergic state. Both extremes have detrimental effects on attention. Comparable nonlinear effects have already been reported in pharmacological studies on the effects cholinergic agonists on attention.

## Introduction

Neurophysiological experiments demonstrated that successful cue detection specifically elicits cholinergic transients in the medial prefrontal cortex (Parikh et al. 2007). In Alzheimer's dementia (AD), which is frequently accompanied by cholinergic deficiency, attention and executive ability decline during early disease stages (White et al. 1977; Haxby et al. 1990). Acetylcholine‐esterase (AChE) inhibitors are thought to delay cognitive decline, but also to improve attentional deficits in individuals at high risk of developing dementia (Winblad et al. 2001; Salloway et al. 2004).

The magnocellular cell clusters in the basal forebrain are the main origin of cholinergic fibres to the cortex, which in its main part compromises the Ncl. basalis of Meynert (NBM or CH4, Mesulam et al. 1983). Some authors describe another cell cluster in the primate brain projecting to the cortex, the Nucleus subputaminalis of Ayala (NSP), although other authors refer to it as a part of the NBM or CH4 (Simić et al. 1999; Boban et al. 2006; Zaborszky et al. 2008).

Selective lesioning of the NBM in rats leads to attentional deficits (Harati et al. 2008). Studies with the objective to investigate the function of the NBM in humans are mainly restricted to research on AD. In AD as well as mild cognitive impairment (MCI), where its degeneration is thought to play a key role, smaller NBM volume predicts cognitive decline (Grothe et al. 2010, 2013).

Whereas the NBM is thought to be the main origin of cholinergic innervation to the whole cortex, the NSP is thought to project to frontal and cingular areas, brain regions that are especially involved in performance of the Stroop‐Task (Mesulam et al. 1983; Simić et al. 1999; Banich et al. 2000). The NSP is thought to be human‐specific and may thus play a pivotal role for higher cognitive functions (Simić et al. 1999). Studies on ageing populations have reported an association of the NSP with IQ, but no specific hypotheses about its function in young individuals have yet been made (Wolf et al. 2014).

In this study we hypothesized that NBM volumes predict cognitive performance, in particular in attention and executive function in young individuals, referring to evidence from fundamental and clinical studies. We further hypothesized that the NSP might have a similar relevance for higher cognitive functions.

Although we had no specific a priori hypothesis on interactions of the cholinergic system including smoking status with demographic variables, we considered them potentially relevant with regard to a role of the NBM for cognitive decline during healthy ageing and neurodegenerative diseases (Fratiglioni et al. 1991; Wolf et al. 2014).

## Materials and Methods

### Participants

We investigated a subsample of *N* = 38 healthy subjects (*N* = 20 females) from the German multi‐centric cohort study “Genetics of Nicotine Dependence and Neurobiological Phenotypes”, who underwent additional neuroimaging investigations (for details see Lindenberg et al. 2011). All participants were randomly selected from the general population. All participants from our subsample were recruited at the Helmholtz Research Centre in Jülich.

Subjects with a history of medical, neurological or axis 1‐psychiatric illness (according to DSM‐IV) or alcohol‐/drug abuse within the last 6 months as assessed by medical interview and examination, routine laboratory tests, an electrocardiogram, drug screening and standardized psychiatric interview were excluded from the study.

All participants gave written informed consent. The study was approved by the local ethics committee and conducted according to the declaration of Helsinki.

Mean age was 33.5 ± 11 years (range: 19–55 years). Median school and professional education was 15 years (range: 9–22 years).

Only never‐smokers (*N* = 18) and current smokers (*N* = 20) according to DSM‐IV were included in this study. Never‐smoking status was defined by lifetime consumption of less than 20 cigarettes. Smokers were included if their score in the Fagerström test for nicotine dependence (FTND) was at least 4. Further details on smoking behavior in our study sample are given in the supplement.

Results of the TMT Part A were available for all the participants, but for TMT Part B and the Stroop‐Task, results were missing for one individual.

### Neuropsychological assessment

Trail‐Making‐Test (TMT) Part A and B as well as the Stroop‐Task were administered to each participant by research assistants as part of a comprehensive test battery taking at maximum 75 min for completion. Assessment took place from 10:00 am to 01:00 pm. Both smokers and never‐smokers were assigned equally to the mid‐morning or midday test session to avoid bias due to an effect of the circadian rhythm on cognitive performance. To avoid nicotine withdrawal effects on test performance in smokers, tests were completed within 3 h after the last cigarette.

The TMT Part A demands the participant to sequentially search and connect numbers, which are distributed on the test sheet in a semi‐randomized manner. Part B additionally requires connecting a set of letters in alphabetical order, switching attention by alternating between numbers and letters. Time for completion of the tests was measured (Reitan 1979).

During the Stroop‐Task the participant is presented color words printed in colored ink and asked to name the color of the letters. We subtracted the time needed to name the color of line from the time needed to name the color of incongruent words to calculate a measure of the interference effect. The incongruent stimuli (e.g. the word “red” printed in green) demand the subject to concentrate attention selectively on a single feature of the stimulus (Stroop 1935; Bäumler 1985).

Trail‐Making Test is a common measure of visual search, and especially Pt. B demands additional attentional resources, whereas the Stroop‐Task is a common paradigm in research on selective attention (Crowe 1998; Banich et al. 2000). Stroop‐Task as well as TMT Pt. B have been used to detect early decline of attentional deficits in progression of Alzheimer's dementia (Haxby et al. 1990).

### Image acquisition

T1‐weighted, 3D structural scans (magnetization prepared rapid gradient echo: repetition time/echo time = 2250/3.03 msec, flip angle = 9°, 176 sagittal slices, field of view = 200 × 200 mm, 64 × 64 mm matrix, voxel size = 1 × 1 × 1 mm) of the participants were acquired using a 3T scanner (TIM‐Trio, Siemens, Erlangen, Germany). All scans were acquired with the same MR scanner at the Helmholtz Research Centre in Jülich.

### Image processing and volume measurement

The volumes of the NBM as well as its subregions were quantified using a validated tissue probability map of cholinergic cores in the basal forebrain (Butler et al. 2014). For a detailed description of the creation of the probabilistic map, we refer to Zaborszky et al. (2008). Ten brains of body donors with no record of neurological or psychiatric disease (five female, age range 37–75 years) were obtained at autopsy and fixed in formaldehyde or Bodian fixative. MR images of the brains were obtained using a 1.5 T Siemens Magnetron SP scanner and a 3D Flash sequence. A modified silver staining method of Gallyas was used for histological delineation of cholinergic cell groups. The cell groups were traced out in the MR images of the subjects and combined into a probabilistic map by normalizing all brain scans to the single subject T1‐weighted MNI reference brain. The probabilistic map distinguishes four different cell clusters which were specified with a modified version of Mesulam's nomenclature. A voxel was assigned to a structure which it represented in at least 40% of the brains. In case the cell clusters from different brains showed overlap in one voxel, the voxel was assigned to the structure with the highest probability.

Image processing was carried out using SPM8 (Wellcome Trust Centre for Neuroimaging, http://www.fil.ion.ucl.ac.uk/spm/software/spm8/) in a MATLAB R2013a environment (The Mathworks, Inc., Natick, MA).

The images were manually aligned to each other and partitioned into grey and white matter using the SPM segmentation routine. Subsequently, the DARTEL Toolbox was applied to create a mean template from all images and calculate flow fields. DARTEL was run a second time to calculate the flow field needed to match the reference brain (http://www.bic.mni.mcgill.ca/ ServicesAtlases/Colin27) used by Zaborszky et al. to the template.

The probability map was then warped to fit the image of each participant. As such, a deformation was applied to each individual image composed of the flow field of the reference brain in a backward deformation and the flow field of each individual image in a forward deformation.

In this way individual tissue maps of each participant were created. Finally, 3DSlicer 4.3.0 (http://www.slicer.org/) was used to calculate the volume of each labelled region in the map.

To achieve volume normalization of the subregions (*V*_norm_), we calculated the quotient of core volume (*V*_CH_) and total brain volume (*V*_tB_). Subsequently, this quotient was multiplied with the average volume of all brains (*V*_avB_) to scale each subregion volume to an average‐sized brain: *V*_norm_ *= *(*V*_CH_/*V*_tB_) *× V*_avB_.

### Statistical analysis

Our probabilistic map distinguishes two subregions of the basal forebrain with projections to the cortex. These are named CH4 and CH4p according to a modified version of Mesulam's nomenclature, first introduced by Zaborszky et al. (2008). CH4 corresponds to the Nucleus basalis Meynert, but CH4p also covers the Nucleus subputaminalis of Ayala.

Since the normalized volumes of the four cholinergic subregions were expected to correlate with each other, we decided to design a general linear model for the effect of each subregion volume (CH4 and CH4p in each hemisphere) on each of the three test scores, yielding twelve analyses in total. *P* < 0.05 was set as the level of significance. Since the three test scores were correlated, we report significant main effects after a Bonferroni‐correction for testing four different subregions. Performance in the TMT Part A correlated with the TMT Part B at *R* = 0.653 (*P* < 0.001) and with the Stroop‐Effect at *R* = 0.468 (*P* = 0.004).

Age (in years), sex and education (school and professional, measured in years) were included in each model as covariates. Interactions of the cholinergic nuclei with these covariates were considered in the model if they showed at least trend for significance at *P* < 0.1. To minimize effects of multicollinearity, all independent variables were centered around their mean. Male and female sex were coded as 0.5 and −0.5, respectively. Additionally, the variance inflation factors were checked if they exceeded a threshold of 2.5. The literature considers a value of >5 as critical (Urban and Mayerl 2011).

During inspection of partial regression plots we found evidence for a quadratic rather than a linear effect of the left CH4 volume on performance in the TMT Part A and B. Referring to a report on the finding that hypoperfusion of the left hemisphere is associated with postoperative low performance in the Trail‐Making‐Test, we considered a focus on this subregion appropriate (Messerotti Benvenuti et al. 2012; Zanatta et al. 2012). Holley et al. (1995) modulated the activity of cholinergic basal forebrain cells in a rat model of sustained attention using a GABA_A_‐receptor antagonist. They report that an “increase in false alarms may model a critical component of the cognitive dysfunctions that are associated with abnormally high activity of cortical cholinergic inputs” with the implication of an inverse u‐shaped effect of cortical acetylcholine on attention. We thus performed a quadratic regression analysis for the effect of the left CH4 volume on TMT performance.

In a post hoc exploratory analysis, we also considered an effect of smoking on test performance and relevant core volumes (see supplement).

We used partial regression plots to assess the direction of main effects and report B‐coefficients from the general linear model equation to interpret the effects of interaction terms. When an interaction term of core volume with a covariate had the same direction like the main effect of core volume, this indicated a stronger effect of the core volume on test performance for larger values of the confounding variable.

Before analysis, data were checked for normal distribution using the Kolmogorov–Smirnov‐test and the assumption of normal distribution was accepted if *P* > 0.05. Homoscedasticity was visually controlled by plotting standardized residuals against standardized predicted values.

IBM SPSS Statistics 21 (IBM Corporation, Armonk, NY) was used for all statistical analyses.

## Results

**Figure 1:**
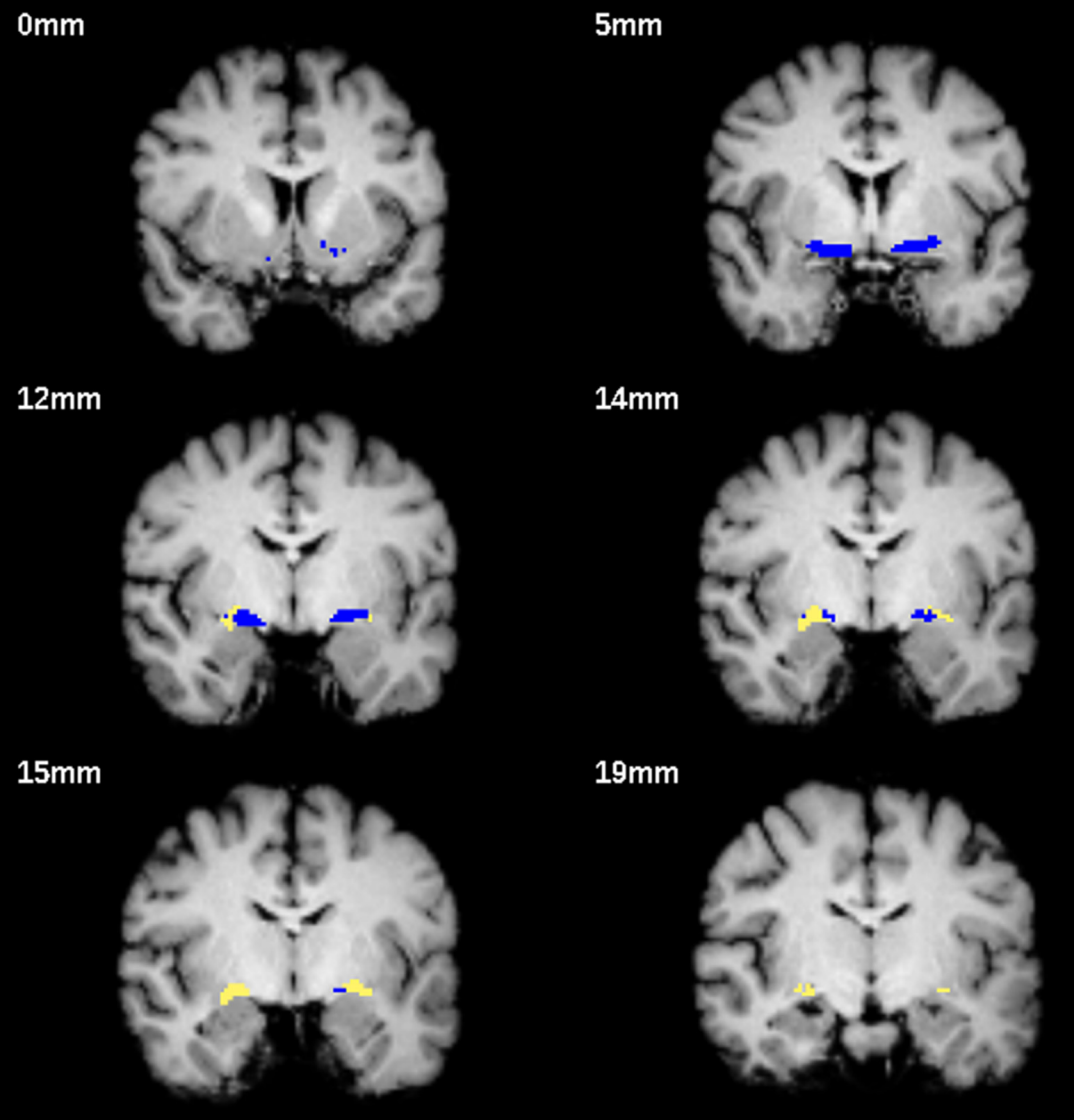
Segmentation example for one brain. The blue ROI refers to CH4, CH4p is shown in yellow. Slices were chosen with the intention to show that CH4p covers a cell cluster often referred to as the Nucleus subputaminalis of Ayala which is rostrolateral extension of the NBM (at 12, 14 and 15 mm). Slice positions are indicated with reference to the most anterior slice on the top left.

Segmentation results for one subject are shown in Figure 1. According to the Kolmogorov‐Smirnov‐test, we could not reject the assumption of normal distribution for test performance (p_TMT‐A_ = 0.976, p_TMT‐B_ = 0.728, p_Stroop_ = 0.336). VIFs were below the threshold of 2.5 in all analyses.

**Figure 2:**
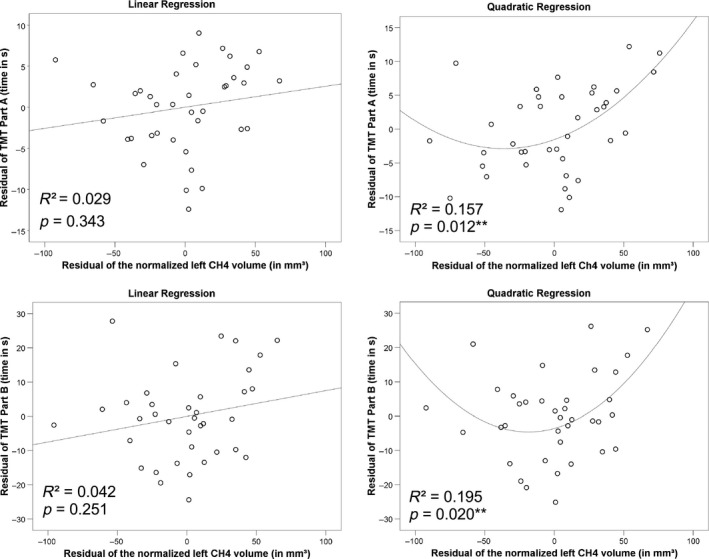
Effects of the left CH4 volume on Trail‐Making Test (TMT) performance analysed in a linear (left) and quadratic (right) regression. The proportion of variance in TMT performance explained exclusively by the left CH4 volume is shown on the bottom left of each plot (R^2^). ** indicates a significant corrected P‐value for the quadratic term. The adjusted R^2^ for each test was larger for the quadratic (adj. R^2^_TMT‐A_ = 0.361, adj. R^2^_TMT‐B_ = 0.571) than the linear model (adj. R^2^_TMT‐A_ = 0.332, adj. R^2^_TMT‐B_ = 0.423), indicating an improvement of the model by using quadratic regression.

The linear models showed no significant main effects of the cholinergic subregions on TMT or Stroop‐Task. However, significant interactions of the left CH4 region and sex were found for all three tests (Stroop‐Task and TMT Part A and B). Sex moderated the effect of the right CH4 region on performance in TMT Part A. Details of main effects of subregion volumes on test performance as well as interactions with covariates are shown in Table 1. Partial regression plots pointed to a quadratic relationship of the left CH4 volume and performance in the TMT (see Fig. 2).

**Table 1:** Linear effects of normalized cholinergic subregion volumes on task performance

Independent variable	TMT Part A B ± SE (*P*)	TMT Part B B ± SE (*P*)	Stroop‐effect B ± SE (*P*)
Left CH4	0.026 ± 0.027 (0.343)	0.075 ± 0.064 (0.251)	0.034 ± 0.047 (0.484)
Left CH4 × Sex	0.162 ± 0.056 (0.007^a^)	0.380 ± 0.127 (0.005^a^)	0.195 ± 0.090 (0.038^a^)
Left CH4 × Age	−0.003 ± 0.002 (0.077)	– (n.s.)	– (n.s.)
Right CH4	0.025 ± 0.029 (0.399)	0.117 ± 0.079 (0.149)	0.015 ± 0.052 (0.782)
Right CH4 × Sex	0.161 ± 0.054 (0.006^a^)	– (n.s.)	– (n.s.)
Left CH4p	−0.037 ± 0.089 (0.683)	−0.184 ± 0.235 (0.439)	−0.023 ± 0.141 (0.874)
Right CH4p	−0.003 ± 0.057 (0.958)	0.048 ± 0.147 (0.744)	−0.036 ± 0.089 (0.691)

TMT, Trail‐Making Test.

a Indicates any effect significant at *P* < 0.05 without Bonferroni correction, (n.s.) indicates an insignificant term which was rejected from the model. Unstandardized B‐coefficients with standard errors (SE) as well as the respective *P*‐values are also shown.

The detailed analysis of a quadratic relationship of the left CH4 and the TMT revealed significant quadratic and linear effects of this subregion on Part A (Fig. 2). The latter was moderated by age (Table 2). Performance in Part B was only significantly influenced by the squared term, and the linear effect was moderated by age (Table 3 and Fig. 2).

**Table 2:** Effects of normalized left CH4 volume and covariates on TMT Part A in the quadratic regression analysis

Independent variable	B‐coefficient ± SE	Standardized *β*‐coefficient	*P*
Squared volume	0.001 ± 0.000	0.534	0.012**
Volume	0.062 ± 0.023	0.418	0.040**
Volume × Age	−0.005 ± 0.002	−0.377	0.022*
Sex	−4.058 ± 2.130	−0.300	0.066
Age	0.076 ± 0.093	0.121	0.421
Education	−0.813 ± 0.360	−0.312	0.031*
Intercept	23.153 ± 1.214		<0.001*

TMT, Trail‐Making Test; SE, standard error.

*Indicates any effect significant at *P* < 0.05 without correction, whereas **indicates a significant effect of core volume on test performance after Bonferroni correction.

**Table 3:** Effects of normalized left CH4 volume and covariates on TMT Part B in the quadratic regression analysis

Independent variable	B‐coefficient ± SE	Standardized *β*‐coefficient	*P*
Squared volume	0.004 ± 0.001	0.507	0.020**
Volume	0.116 ± 0.058	0.302	0.216
Volume × Age	−0.012 ± 0.004	−0.389	0.006*
Volume × Sex	0.249 ± 0.135	0.301	0.076
Sex	0.115 ± 5.213	0.003	0.983
Age	0.057 ± 0.203	0.035	0.773
Education	−1.099 ± 0.853	−0.161	0.761
Intercept	51.050 ± 2.704		<0.001*

TMT, Trail‐Making Test; SE, standard error.

*Indicates any effect significant at *P* < 0.05 without correction, whereas **indicates a significant effect of core volume on test performance after Bonferroni correction.

We further analysed the relationship of CH4 volume and smoking. CH4 volumes did not differ between smokers and never‐smokers. The effect of the left CH4 volume on TMT performance was not restricted to either the group of smokers or never‐smokers (see supplement for details.)

## Discussion

### Effects of NBM volumes on attention and executive functions

In the present study we found a non‐linear effect of the left CH4 volume on performance in the TMT, but not on the Stroop‐Effect. This may indicate a comparatively higher sensitivity of the TMT to the effects of central cholinergic system parameters on cognition. Notably, the cohort study from which our sub‐sample was drawn showed that smokers performed worse on the TMT, but not on the Stroop‐Task (Wagner et al. 2013).

The effect of the volume on performance was u‐shaped (quadratic), predicting good performance for individuals with core volumes close to the average, whereas extremely small or large volumes were associated with poor results in the TMT. The finding that small cholinergic core volumes are associated with poor performance is in line with studies on the cholinergic system in ageing and neurodegenerative diseases (e.g. Alzheimer's disease) as well as animal studies on NBM lesions (Harati et al. 2008; Grothe et al. 2013; Wolf et al. 2014). We assume that a small volume might be associated with a relative hypocholinergic state in healthy young individuals. On the other hand, we suggest that an extremely large volume predisposes an individual for hypercholinergic states. Studies on AChE inhibitor application in healthy participants showed a cognitive impairment rather than an enhancement and a bell‐shaped dose‐response curve has been proposed for the effects of several cholinergic receptor agonists on cognition (Kennedy et al. 2003; Kitagawa et al. 2003; Beglinger et al. 2004, 2005). Holley et al. (1995) modulated the activity of cholinergic cells in the basal forebrain of rats during a cue detection task using an GABA_A_‐receptor agonist or antagonist. They found that inhibition of ACh release decreased the number of detected cues, whereas dis‐inhibition of the cholinergic cells increased the number of false rejection of distractor cues.

Several studies showed a lateralization effect toward the left hemisphere during performance of the TMT Part B (Moll et al. 2002; Zakzanis et al. 2005). These studies suggest a relevance of left frontal, cingular and motion‐related regions to set shifting. These areas are structurally and functionally connected to the CH4 region, and thus an effect of its volume on the TMT seems reasonable (Mesulam et al. 1983; Li et al. 2014). The CH4p region is thought to project to the speech‐area in the inferior frontal gyrus as well auditory areas in the temporal lobe, which explains the low impact of its volume on visually attention‐demanding tasks (Mesulam et al. 1983; Simić et al. 1999; Zaborszky et al. 2008). Both parts of the TMT, especially Part A, require visual search (Crowe 1998). Although the attention network has been thought to be right‐lateralized, activation in circumscribed regions in left frontal and parietal areas has been attributed to several subdomains of visual search (Weidner et al. 2009). Additionally, performance in the TMT has been found to be specifically affected by hypoperfusion of the left hemisphere, which is in line with our finding of the left CH4 region specifically affecting TMT performance (Messerotti Benvenuti et al. 2012; Zanatta et al. 2012).

The effect of the left CH4 on TMT performance was moderated by age, and our data suggest, that with increasing age, even a large core volume would less likely cause impairment of test performance. Thus, the risk of shifting into a hypercholinergic state is decreased during ageing. This is in line with the epidemiology of AD, a disease of elderly patients, which is associated with a hypocholinergic state (Fratiglioni et al. 1991). Decreasing ACh synthesis and release by the cholinergic neurons might be accountable for this finding, since choline‐acetyltransferase and ACh‐esterase activity have been shown to decrease early during the lifespan (Perry et al. 1992; Sparks et al. 1992).

Although the linear model did not show a main effect of cholinergic subregions on attention in the first approach, results suggest an interaction of subregion volumes with sex. Men and women have been suggested to use different strategies and cognitive systems in tasks of visual attention, which may be differentially be influenced by acetylcholine (Rubia et al. 2010). The direction of the interaction effect suggested that among individuals with above‐average CH4 volumes, women would outperform men in attention demanding tasks. Since men recruit especially left‐sided cortical regions involved in saliency‐dependent processes for attention allocation, they might be at risk for high levels of ACh to induce false‐rejection errors (Rubia et al. 2010).

### Limitations

Our study sample included regular smokers in addition to never‐smokers. Since smokers have cognitive deficits especially in the domain of attention, the generalizability of our results may be limited (Wagner et al. 2013). To estimate the extent to which the inclusion of smokers in our study affects our results, we performed an additional analysis, which is described in detail in the supplement of this article. The results from our study suggest that the high fraction of smokers does not affect the generalizability of our results.

The TMT was part of a comprehensive test battery and we do not know how preceding tests interfere with its assessment. Sustaining attention during the whole neuropsychological examination might facilitate a hypercholinergic state by ACh accumulation especially in individuals with a large nucleus.

TMT and Stroop‐Task are common tests used for the examination of attention, but include different subcomponents of attention, such as visual search in TMT Parts A and B, but divided attention and set‐shifting especially in TMT Part B (Crowe 1998). These subcomponents are also constituent part in other cognitive domains, which could be affected by acetylcholine, but are not attention‐related in the strict sense.

## Conclusions

We describe an association of an NBM subregion volume with performance in tests of attention and executive functions. Cholinergic innervation seems to be especially important for performance in visual search tasks. Extremely small as well as extremely large volumes are associated with poor test performance, indicating that either hypocholinergic or hypercholinergic states impair cognition.

## Conflict of interest

This publication is part of Florian Lammers’ doctorate. All authors have no conflicting financial interests.

## Supporting information


**Table S1**. Demographic data of smokers and never‐smokers.
