# Supplementary material for: Effects of Ncl. Basalis Meynert volume on the Trail‐Making‐Test are restricted to the left hemisphere
Source: Brain Behav. 2015 Dec 29;6(1):e00421. doi: 10.1002/brb3.421 (PMC4834944; doi:10.1002/brb3.421)
Supplement: Supplementary file 1 — Table S1. Demographic data of smokers and never‐smokers. [file BRB3-6-e00421-s001.pdf]

SUPPLEMENT

## 1 Nicotine dependence and the cholinergic system

Affection of the central cholinergic system is considered to play a role in the pathogenesis of nicotine dependence (Winterer et al. 2010). An fMRI study on visual attention revealed increased activation of the basal forebrain in smokers, suggesting an affection of the NBM in particular (Vossel et al. 2011). We considered it therefore possible, that the effect of NBM volume on attention is modulated by a history of nicotine dependence.
On the other hand, the sample composition in our study (heavy and never-smokers) might affect the generalisability of our results.
The study, from which this sub-sample was drawn, has shown that smokers have cognitive deficits that are not attributable to withdrawal. These deficits especially concern the performance in the TMT and other tests of visual attention (Wagner et al. 2013).
We performed additional statistical analyses to address the questions if (1) smoking habits affects CH4 volumes and (2) the effect of the left CH4 volume on test performance interacts with the effect of nicotine dependence.

## 2 Baseline data according to smoking habits

Baseline data did not differ significantly between smokers and never-smokers. Table S1 provides details about the demographic data in both groups.
Smokers consumed at least two and up to 35 cigarettes per day with an average of 15.5 at the time of data acquisition. They reported a smoking history of up to 41.2 packyears, with a mean of 12.5±13.9 packyears.
Blood samples for measurement of cotinine plasma levels were taken at the end of the test day, two hours after the participant had smoked a second cigarette. Plasma samples were kept at -80°C pending the chemical analysis. Mean cotinine levels in smokers were 85.2±96.3ng/mL, levels in never-smokers were below the detection threshold.

**TABLE S1: Demographic data of smokers and never-smokers.**

| | Smokers | Never-smokers | p | Test procedure |
|---|---|---|---|---|
| Age (mean±SD) | *35.65±11.07yrs* | *31.17±10.64yrs* | *0.212* | Student's t-test<br>df=36, t=-1.270 |
| Sex (female/male) | *8/10* | *12/8* | *0.746* | Chi-Square-test<br>df=1, $\chi$=0.105 |
| Years of Education (median, range) | *15, 9-22yrs* | *15.5, 11-18yrs* | *0.906* | Mann-Whitney-U-test<br>U=176 |

## 3 Statistical Analysis

The left and the right normalised CH4 volume were used as the dependent variables in two analyses of covariance (ANCOVA) of which each included current smoking (yes/no) as the covariate of interest, and age and sex as covariates of no interest. Interactions among the independent variables were rejected from the model, if they were insignificant at $p \geq 0.1$.
To check effects of smoking on our analysis of TMT performance, we added smoking (yes/no) and the interaction effect of smoking and left CH4 volume and squared left CH4 volume to the regression model. Smokers were coded as 0.5, never-smokers as -0.5.
We report significant results at an uncorrected threshold of $p<0.05$.

## 4 Results

Smoking affected neither the left (F=0.006, df=33, p=0.940), nor the right CH4 volume (F=0.225, df=33, p=0.638).
For Part A of the TMT, there was a significant interaction of smoking and the squared volume (B=-0.002±0.001, p=0.031), but the main effects of the volume also remained significant (quadratic: B=0.001±0.000, p=0.003; linear: B=0.069±0.022, p=0.004). There was no significant interaction of the linear effect of left CH4 volume with smoking (p=0.578).
For Part B of the TMT, we found no significant interactions of smoking and the left CH4 volume (quadratic: p=0.165; linear: p=0.482). In the regression analysis of the TMT Part B, the VIFs exceeded the threshold of 2.5 (3.118 maximum).

## 5 Conclusions

The results of our additional analyses showed no effect of nicotine dependence on the normalised CH4 volumes.
Results from the analysis of interaction terms of smoking and the left CH4 volume are somewhat difficult to interpret: There was an interaction of the quadratic volume effect and smoking on TMT Part A, indicating that volume differences in smokers affected performance in Part A less than in never-smokers, which might reflect decreased sensitivity of smokers to ACh release. Decreased ACh sensitivity has been suggested to predispose to nicotine dependence due to its effect on cognitive performance (Ernst et al. 2001). Nevertheless, we could not reproduce this finding in the analysis of TMT Part B, although one would expect that performance in both tests depends on similar cognitive mechanisms. To fully elucidate this point will need further studies. Since VIFs slightly exceeded the threshold value in the analysis of TMT Part B, multicollinearity might have affected our results.
Since smokers did not differ in regards of CH4 volumes, and interactions of left CH4 volume that could affect TMT performance were either insignificant (Part B) or did not affect the significance of main effects (Part A), we conclude that our results can be generalised for other populations.

## 6 References

Ernst, M., Heishman, S.J., Spurgeon, L., London, E.D., 2001. Smoking history and nicotine effects on cognitive performance. Neuropsychopharmacology 25, 313–319. doi:10.1016/S0893-133X(01)00257-3

Vossel, S., Warbrick, T., Mobascher, A., Winterer, G., Fink, G.R., 2011. Spatial and sustained attention in relation to smoking status: behavioural performance and brain activation patterns. J. Psychopharmacol. (Oxford) 25, 1485–1495. doi:10.1177/0269881110391830

Wagner, M., Schulze-Rauschenbach, S., Petrovsky, N., Brinkmeyer, J., von der Goltz, C., Gründer, G., Spreckelmeyer, K.N., Wienker, T., Diaz-Lacava, A., Mobascher, A., Dahmen, N., Clepce, M., Thuerauf, N., Kiefer, F., de Millas, J.W., Gallinat, J., Winterer, G., 2013. Neurocognitive impairments in non-deprived smokers--results from a population-based multi-center study on smoking-related behavior. Addict Biol 18, 752–761. doi:10.1111/j.1369-1600.2011.00429.x

Winterer, G., Mittelstrass, K., Giegling, I., Lamina, C., Fehr, C., Brenner, H., Breitling, L.P., Nitz, B., Raum, E., Müller, H., Gallinat, J., Gal, A., Heim, K., Prokisch, H., Meitinger, T., Hartmann, A.M., Möller, H.-J., Gieger, C., Wichmann, H.-E., Illig, T., Dahmen, N., Rujescu, D., 2010. Risk gene variants for nicotine dependence in the CHRNA5-CHRNA3-CHRNB4 cluster are associated with cognitive performance. Am. J. Med. Genet. B Neuropsychiatr. Genet. 153B, 1448–1458. doi:10.1002/ajmg.b.31126
